# Editorial: Global excellence in inflammatory diseases: North America 2021

**DOI:** 10.3389/fimmu.2023.1245827

**Published:** 2023-07-06

**Authors:** Linda L. Kusner, Ravi S. Misra, Rudolf Lucas

**Affiliations:** ^1^ The Laboratory for Myasthenia Gravis Research, Pharmacology and Physiology, The George Washington University, Washington, DC, United States; ^2^ Department of Pediatrics-Neonatology, University of Rochester-Golisano Children’s Hospital, Rochester, NY, United States; ^3^ Vascular Biology Center, Department of Pharmacology and Toxicology, Augusta University, Augusta, GA, United States; ^4^ Division of Pulmonary, Critical Care and Sleep Medicine, Department of Medicine, Medical College of Georgia at Augusta University, Augusta, GA, United States

**Keywords:** e-cigarette, olfactory receptors, granulopoiesis, pulmonary hypertension, autoimmune myasthenia gravis, melanoma, interstitial fibrosis and tubular atrophy

This manuscript is an introductory editorial to the special topic “*Global Excellence in Inflammatory Diseases: North America 2021*”.

This Research Topic assembles four original and three review contributions by leaders in the field of inflammatory diseases, situated in North-America. As summarized in **Table 1**, topic areas covered are:

1. an update on the pathophysiology of inflammatory diseases [e.g. atherosclerosis, melanoma, bacterial pneumonia and sepsis, HIV-associated pulmonary hypertension (PH), interstitial fibrosis and tubular atrophy (IFTA), myasthenia gravis and e-cigarette/Influenza A virus-induced acute respiratory distress syndrome (ARDS)].
2. novel receptors regulating inflammation [e.g. olfactory receptors in macrophages]
3. cells mediating inflammatory diseases [e.g. CD16^+^ monocyte/CD8^+^ T cell interactions in melanoma, hematopoietic stem and progenitor cells (HSPCs) in emergency granulopoiesis, and AChR-specific T_H_ cells in Myasthenia Gravis]
4. inflammatory and anti-inflammatory mediators (e.g. Octanal in atherosclerosis, CCL21 in melanoma, C/EBP-β and CXCL1 in pneumonia, EMAP II in HIV-associated PH, Beclin-1 in IFTA, NFκB2 in MG and aerosolized nicotine and MUC5AC in IAV infection)
5. pathways involved in disease progression (e.g. STING and ISR pathways in IFTA)
6. translational mouse models for studying inflammatory diseases (e.g. *E2^-/-^* mice in melanoma, *Cxcl1^-/-^* mice in bacterial pneumonia, *NSG* (humanized) mice in HIV/PH, *Becn^1F121A/F121A^* mice in IFTA and AChR-immunized C57Bl/6, SJL, AKR mice in MG.

**Table 1:** Summary of inflammatory diseases and of the cell types, mediators, receptors and pathways involved, as well as mouse models used.

Topic Contribution	Disease	Cell types	Mediators Pathways	Binding partners	Mouse Models
Orecchioni et al. ^R^	Atherosclerosis	Macrophages	Octanal Lactate	Olfactory receptors	*Ldlr^-/-^*
Padgett et al. ^O^	Melanoma	CD16^+^ monocytes CD8^+^ T cells	CCL21	CCR7	*E2^-/-^*
Paudel et al. ^R^	Bacterial pneumonia	HSPCs Neutrophils	C/EBP-β IL27 CXCL1	TLR2 TLR4	*C3H/HeJ* *Cxcl1^-/-^*
Rodriguez-Irizarry et al. ^O^	HIV/Pulmonary hypertension	VSMCs Endothelial cells	EMAP II	Endothelin receptor A	*NSG*
Lopez-Soler et al. ^O^	Interstitial Fibrosis and Tubular Atrophy (IFTA)	Fibroblasts Tubular cells Endothelial cells	Beclin-1 STING/ISR pathways	VPS15 VPS34 UVRAG ATG14	*Becn^1F121A/F121A^*
Huda ^R^	Autoimmune Myasthenia Gravis	Monocytes/MΦ AChR-spec T_H_ Dendritic cells B cells	AChR-specific auto-Ab IL-4, IFN, NFκB2	AChR TLR-3,7,9	AChR-immunized C57Bl/6, SJL, and AKR
Maishan et al. ^O^	IAV pneumonia ARDS	AT1/2 Endothelial cells	Nicotine VG PG MUC5AC	nAChR	IAV-infected C57BL6

R, review; O, original; HSPCs, hematopoietic stem and progenitor cells; VSMCs, vascular smooth muscle cells; AChR, acetylcholine receptor; EMAP II, endothelial monocyte activating polypeptide II; IAV, Influenza A virus; MUC5AC, Mucin 5 subtype AC; nAChR, nicotinic acetylcholine receptor.

The majority of manuscripts briefly discuss emerging therapeutic approaches aimed at mitigating inflammatory diseases, along with the associated challenges.

Orecchioni et al. elaborate in their concise review on their recent findings that olfactory receptors (ORs), which bind odorous ligands, are not only expressed in the nasal olfactory epithelium, but also in extranasal macrophages. The review discusses how activation of different ORs in macrophages with either octanol on the one hand or with lactate on the other hand can aggravate inflammasome activation in atherosclerosis or dampen the immune response in cancer, respectively. In view of these landmark findings, the authors advocate the need for deorphanizing more ORs and determining the sources of their ligands, which can foster the development of novel anti-inflammatory therapies.

Padgett et al. contribute with an original study aiming to investigate how the little known non-classical CD16^+^ monocytes influence the CD8^+^ effector T cell-mediated anti-tumor response in mice injected with the B16F10-OVA melanoma cell line. To do so, the authors compare tumor progression between wild type mice and an in-house developed *E2^-/-^* mouse strain, which specifically lacks non-classical monocytes following depletion of the Nr4a1 E2 super enhancer subdomain, while preserving classical monocyte frequencies and macrophage function.

Paudel et al. provide a comprehensive review of the emerging concepts the hematopoietic system uses during acute inflammatory diseases (e.g. pneumonia and sepsis) to rapidly switch from steady state to emergency granulopoiesis. This switch is necessary to augment neutrophil production in the bone marrow and to release them to the blood, from where they are recruited in large numbers to peripheral organs to cope with demand. However, when recruited in excessive numbers, neutrophils can induce severe organ injury (like in the lungs). The review also reassesses the well-established cellular and molecular mechanisms associated with granulopoiesis and as such fosters the reader’s understanding of the mechanisms governing granulopoiesis, which is critical to modulate the detrimental inflammatory process in numerous organs following infectious assault.

Thanks to anti-retroviral therapy, HIV patients have a comparable life span to non-infected subjects, but they unfortunately have a higher tendency to develop life-threatening pulmonary hypertension (PH), due to the chronic inflammation in their lungs. Currently, small animal models combining HIV infection with PH do not accurately represent the human condition. Rodriguez-Irizarry et al. combine HIV infection with the PH-inducing VEGF antagonist SU5416 in humanized *NSG* mice. The main aim is to evaluate whether this represents a valid translational small animal model for PH in HIV patients. The second aim of their studies is to detect inflammatory mediators that are increased upon combining both insults, with a focus on endothelial-monocyte activating polypeptide II (EMAP II).

Unilateral ureteral obstruction (UUO) injury in mice represents a translational model of Interstitial Fibrosis and Tubular Atrophy (IFTA), the most common cause of long-term graft failure following renal transplant, characterized by interstitial fibrosis and the loss of a normal renal architecture. Lopez-Soler et al. present an original study investigating the role for Beclin-1-dependent autophagy in the development of renal fibrosis following UUO in mice. The authors use an elegant approach by comparing several parameters in UUO-injured and uninjured kidney samples, such as fibrosis, autophagy flux, the inflammatory STING pathway and the Integrated Stress Response (ISR) between wild type mice and animals expressing a constitutively active form of Beclin-1.

Myasthenia Gravis (MG) is a neuromuscular autoimmune disorder characterized by chronic fatigue of the eye muscles as well as other skeletal muscles. The muscle weakness occurs primarily as a consequence of the binding of an autoantibody to the acetylcholine receptors, which abrogates normal neuromuscular signal transmission. Huda provides an update on the association and correlation of inflammation with the initiation, progression and severity of MG. The concise review focuses on immune cell interactions (MΦ, T_H_, T_regs_, T_H_17, B cells, DC) the role of immunomodulator molecules in disease development (pro-inflammatory cytokines and chemokines) and critically discusses current therapeutic strategies targeting inflammation in MG (including combination therapy targeting both components, auto-antibody and inflammation).

The use of nicotine-containing e-cigarettes has rapidly increased in recent years. Aerosols produced through the use of e-cigarettes can be immunosuppressive and pro-inflammatory. Influenza A virus infection (IAV) is one of the main causes of viral pneumonia, accompanied by intense inflammation. IAV-induced pneumonia can cause potentially lethal acute respiratory distress syndrome and a good host response to infection is crucial. Maishan et al. provide results from an original study investigating and comparing the effects of, on the one hand aerosolized e-cigarette carrier [vegetable glycerin (VG) and propylene glycol (PG)] and on the other hand aerosolized carrier (VG/PG) with nicotine, on i) the pro-inflammatory response to IAV-induced pneumonia, ii) the transcriptomic response to the pathogen, iii) host defense mechanisms, iv) lung barrier integrity and v) viral clearance during IAV infection. They moreover focus on the presence of Mucin 5 subtype AC (MUC5AC) in the distal airspaces, a compound shown to be protective and to improve viral clearance in IAV infection.

This Research Topic only addresses a selection of the vast subject of inflammatory diseases. For more updates on these and other inflammatory diseases, the authors refer the reader to other recent Research Topic on this topic in *Frontiers in Immunology - Inflammation*, such as “*Advances in Autoimmune Myasthenia Gravis*” (1), “*Autoimmunity and Chronic Inflammation in Early Life*” (2) and “*Insights in Inflammation 2022*” (3).

## Author contributions

RL wrote the original manuscript. LK and RM edited and approved the manuscript.

## References

[1] Punga AR Kusner L Berrih-Aknin S Le Panse R. Editorial: advances in autoimmune myasthenia gravis. Front Immunol (2020) 11:1688. doi: 10.3389/fimmu.2020.01688 32983085 PMC7484602

[2] Misra R Mulligan JK Rowland-Jones S Zemlin M. Editorial: autoimmunity and chronic inflammation in early life. Front Immunol (2021) 12:761160. doi: 10.3389/fimmu.2021.761160 34567015 PMC8458802

[3] Ghezzi P Lucas R Mader S Miossec P Sacre S. Editorial: insights in inflammation: 2022. Front Immunol (2023) 14:1224343. doi: 10.3389/fimmu.2023.1224343 37325656 PMC10264837

